# Static Hot Air and Infrared Rays Roasting are Efficient Methods for Aflatoxin Decontamination on Hazelnuts

**DOI:** 10.3390/toxins9020072

**Published:** 2017-02-21

**Authors:** Ilenia Siciliano, Barbara Dal Bello, Giuseppe Zeppa, Davide Spadaro, Maria Lodovica Gullino

**Affiliations:** 1 Agroinnova—Centre of Competence for the Innovation in the Agro-Environmental Sector, University of Turin, Largo Paolo Braccini, 2 Grugliasco, Turin 10095, Italy; ilenia.siciliano@unito.it (I.S.); marialodovica.gullino@unito.it (M.L.G.); 2 DISAFA—Department of Agricultural, Forest and Food Science, University of Turin, Largo Paolo Braccini, 2 Grugliasco, Turin 10095, Italy; barbara.dalbello@unito.it (B.D.B.); giuseppe.zeppa@unito.it (G.Z.)

**Keywords:** aflatoxins, *Aspergillus flavus*, *Corylus avellana*, fatty acids, thermal treatment

## Abstract

Aflatoxins are a group of secondary metabolites produced by members of *Aspergillus* Section *Flavi* that are dangerous to humans and animals. Nuts can be potentially contaminated with aflatoxins, often over the legal threshold. Food processes, including roasting, may have different effects on mycotoxins, and high temperatures have proven to be very effective in the reduction of mycotoxins. In this work, two different roasting methods—traditional static hot air roasting and infra-red rays roasting—were applied and compared for the detoxification of hazelnuts from Italy and Turkey. At the temperature of 140 °C for 40 min of exposure, detoxification was effective for both roasting techniques. Residual aflatoxins after infra-red rays treatments were lower compared to static hot air roasting. On Italian hazelnuts, residual aflatoxins were lower than 5%, while for Turkish hazelnuts they were lower than 15% after 40 min of exposure to an infra-red rays roaster. After roasting, the perisperm was detached from the nuts and analyzed for aflatoxin contents. Residual aflatoxins in the perisperm ranged from 80% up to 100%. After roasting, the lipid profile and the nutritional quality of hazelnuts were not affected. Fatty acid methyl esters analyses showed a similar composition for Italian and Turkish hazelnuts.

## 1. Introduction

Aflatoxins (AFs) are a group of secondary metabolites produced by members of *Aspergillus* Section *Flavi*—mainly *A. flavus* and *A. parasiticus* [1]—on a variety of food products, such as nuts, grains, and spices [2]. Since 1960, more than 20 aflatoxins have been identified; only four of them—aflatoxin B_1_ (AFB_1_), aflatoxin B_2_ (AFB_2_), aflatoxin G_1_ (AFG_1_), aflatoxin G_2_ (AFG_2_)—occur naturally [3,4]. AFs are a group of difuranocoumarin derivatives named based on their fluorescence under UV-light. AFB_1_ and AFB_2_ have a blue fluorescence due to the difuro-coumaro-cyclopentenone structure, while a six-member lactone ring replacing the cyclopentenone is responsible for the yellow-green fluorescence of AFG_1_ and AFG_2_ [5]. AFB_1_ and AFG_1_ have also an olefinic double bond at the C_8_-C_9_ position, whereas AFB_2_ and AFG_2_ lack this bond. AFs are toxic, mutagenic, teratogenic, carcinogenic compounds implicated in human hepatic and extrahepatic carcinogenesis [6]. AFB_1_ is the most widespread among AFs, and has the most potent carcinogenic effect. In fact, AFB_1_ is the only one classified by International Agency of Research on Cancer (IARC) as a Group 1 carcinogen [7].

Toxicological and epidemiological data, together with occurrence and distribution data, were used to fix limits for food consumption contaminated with mycotoxins. In Europe, thresholds for AFB_1_ and total AFs in hazelnuts for direct human consumption and for use as ingredient in foodstuffs are 5 and 10 μg/kg, specified by the Commission Regulation (EU) No 165/2010, based on scientific opinion of authoritative bodies, such as JECFA (Joint FAO/WHO Expert Committee on Food Additives), WHO (World Health Organization), FAO (Food and Agriculture Organization of the United Nations), EFSA (European Food Safety Authority). Moreover, the Codex Alimentarius Commission has specified 15 μg/kg as limit for peanuts, almonds, shelled Brazil nuts, hazelnuts, and pistachios [8].

Nuts and dried fruits are known to be possibly contaminated with mycotoxins. Different studies on pistachios, almonds, groundnuts, peanuts, chestnuts, and hazelnuts have demonstrated that contamination with aflatoxins often exceeds the legal threshold in commercial samples [9,10,11]. 

Prevention of mycotoxin contamination in the field is the best way to reduce mycotoxins in food and feed, while decontamination is difficult and frequently lowers the quality or quantity of the commercial product. Chemical, physical, and biological approaches for the detoxification of aflatoxins have been reported in previous studies on nuts, and include strategies such as ozone, plasma, UV or gamma irradiation, thermal treatments, or microorganisms [12,13,14,15]. However, most of the current methods are not practical, due to time consumption, nutrition losses, or low detoxification efficiency.

Many nuts are predominantly consumed roasted. Thermal treatments such as roasting lead to chemical changes in carbohydrates, proteins, and fats. The flavor, color, texture, and appearance of hazelnuts are significantly enhanced by roasting, and this process is also used to remove the pellicle of kernels, to inactivate enzymes that cause nutrient loss, and to destroy microorganisms, toxins or allergens [16].

Turkey is the main hazelnut producing country in the world, with 660,000 tons in 2012 [17]. The main cultivar produced in Turkey is “Tombul”, which occupies 29.8% of all production areas, followed by the cultivars “Çakildak” (15.2%), “Mincane” (14.1%), “İncekara” (12.4%), “Palaz” (11.9%), “Foşa” (7.0%), and “Kalinkara” (2.7%). Italy is the second world hazelnut producer, with about 85,000 tons in 2012 [17], and “Tonda Gentile Trilobata” is the main cultivar in Piedmont, Northern Italy (15,000 tons in 2014). Hazelnuts provide an excellent source of energy due to their high oil content of about 60% and several bioactive nutrients. Hazelnut oil has been reported to be the richest source of vitamin E and fatty acids, in particular oleic acid [18]. Among the fatty acids of hazelnut oil, palmitic acid (2.96%–7.40%) is the main saturated fatty acid (SFA). The highest percentage of fatty acids are instead covered by monounsaturated fatty acid (MUFA) as oleic acid (73.1%–90.7%) and polyunsaturated fatty acid (PUFA) as linoleic acid (4.4%–16%), which confer heart and blood beneficial attributes to the hazelnut oil [14]. Moreover, MUFA and PUFA—and particularly linoleic acid—play an important role not only in terms of health, but also in stability, because their oxidation may cause flavor degradation and rancidity. [19]. Fatty acid composition is closely correlated with the stability and degradation kinetics of hazelnuts. In particular, linoleic acid plays the most important role in the stability, and its oxidation may cause flavor degradation and rancidity.

By considering the possibility of aflatoxin contamination on hazelnuts that is often over the legal threshold and the effect of high temperature on mycotoxin reduction, in this work two different roasting methods—traditional static hot air roasting and infrared rays roasting—were compared in the detoxification of hazelnuts from aflatoxins, and their effect on hazelnut quality.

## 2. Results and Discussion

### 2.1. Static Hot Air Treatments

Data of the first trial on hazelnuts roasted with a static hot air roaster are shown in Table 1 for two temperatures (120 °C and 170 °C) at two exposure times (20 and 40 min). Residual aflatoxins after roasting showed a different trend for Italian and Turkish hazelnuts; a decrease was observed at the same temperature with increasing exposure time only for Turkish hazelnuts. With the increase of temperature, differences in reduction were registered, either considering the geographical origin of the hazelnuts or the mycotoxin. In general, the residue was lower in AFB_1_ and AFG_1_ than AFB_2_ and AFG_2_. The influence of temperature was different in both types of hazelnuts used. Turkish hazelnuts showed a decrease in AFs concentration with increasing temperatures and the same exposure time. On the contrary, in Tonga Gentile Trilobata (TGT) hazelnuts, a decrease in the residual concentration was not observed. The decomposition temperature of aflatoxins ranges from 237 °C to 306 °C [20]; in particular, AFB_1_ is stable to dry heating at temperature below its thermal decomposition temperature (267 °C), so the temperatures used during the experiments were below this threshold. Normal home cooking conditions failed to totally destroy AFB_1_ and AFG_1_, and temperatures of approximately 150 °C were necessary to obtain a partial degradation of AFs [21]. In a study on pistachio nuts [22], the reduction of AFs ranged from 17% to 63% and was time and temperature dependent. In coffee beans, a reduction of about 42%–56%—depending on temperature and type of roasting—was achieved [23]. Other parameters seem to influence the rate of AFs destruction: higher moisture content increased AFB_1_ destruction on rice. The presence of water could help the lactone ring opening of AFB_1_ with the formation of a carboxylic group. On both hazelnut types, fatty acid methyl esters (FAMEs) were analyzed (Supplementary Tables S1 and S2), showing that the nutritional composition was not affected at all tested temperatures and exposure times.

### 2.2. Comparison of Static Hot Air and Infrared Rays Roasting

In a second experimental set, a new roasting technology—infrared rays roasting—was compared to static hot air roasting. An intermediate temperature (140 °C) and two exposure times (20 min and 40 min) were used for both methods.

FAMEs analysis showed a similar composition for both Italian (Table 2) and Turkish (Table 3) hazelnuts, and a total of fourteen fatty acids (FAs) were identified. The main FAs were oleic acid, linoleic acid, palmitic acid, and stearic acid. Monounsaturated fatty acids were always higher than polyunsaturated fatty acids and saturated fatty acids. FAs distribution was preserved after all treatments for both types of hazelnuts, both after static hot air and infrared rays treatment. FAs composition is important for nutritional quality and health benefits offered by MUFAs and PUFAs, flavor, kernel texture, and quality [18]. Some minor changes occurred in the FAs profile after both types of roasting. In particular, the content of SFAs remained low, and the content of UFAs that promote health benefits was always high.

The oleic to linoleic acid (O/L) ratio was considered an important criterion to evaluate the kernel quality, and a greater value indicates better oxidative stability [24]. In TGT hazelnuts, the O/L ratio was reduced after all treatments, but the ratio was higher after infrared rays than after static hot air. The degree of unsaturation expressed as iodine value (IV) was higher after static hot air treatments at both exposure times (88.43 and 88.99 instead of 88.34 of raw hazelnuts). After infrared rays treatments at 140 °C for 20 min, a slight IV reduction occurred (87.97 instead of 88.34 of raw hazelnuts).

Turkish hazelnuts (Table 3) showed a similar behavior. The O/L ratio was reduced after all treatments. At 140 °C for 20 min, the O/L ratio was higher after infrared rays than after static hot air (13.34 instead of 12.93). At 140 °C for 40 min, IV was high after static hot air and lower after infrared rays (88.87 and 87.91, respectively, instead of 88.83 of raw hazelnuts).

These results are in agreement with Amaral et al. [25] and Belviso et al. [26], confirming that the lower roasting temperature did not strongly affect the lipid profile and thus the nutritional quality of hazelnuts.

**Figure 1:**
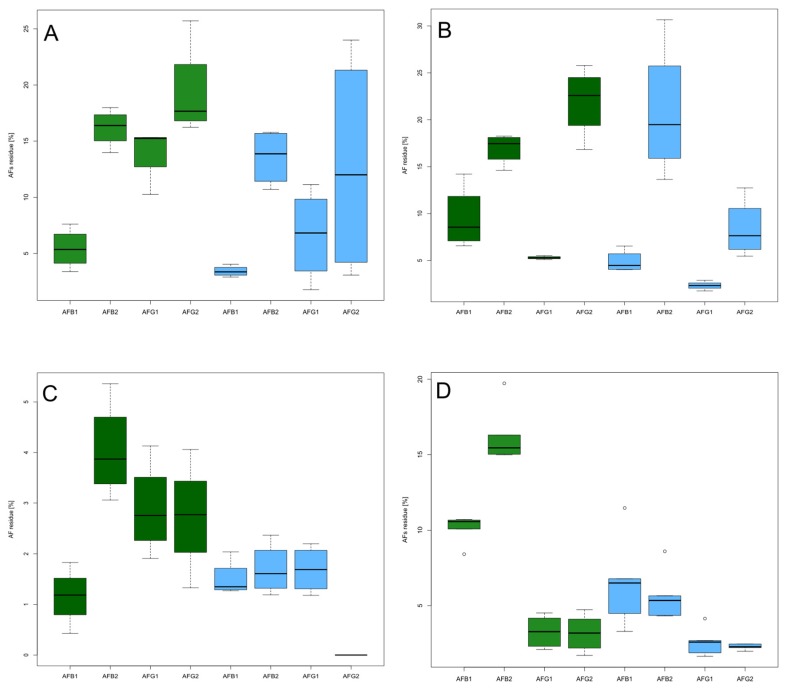
Residual AFB_1_, AFB_2_, AFG_1_, and AFG_2_ after treatments with static hot air roaster compared with infrared rays roaster. Different colors represent different treatments: green, 140 °C for 20 min; blue, 140 °C for 40 min. The treatments were performed on (**A**) Italian TGT hazelnuts roasted with static hot air roaster; (**B**) Turkish hazelnuts roasted with static hot air roaster; (**C**) Italian TGT hazelnuts roasted with infrared rays roaster; (**D**) Turkish hazelnuts roasted with infrared rays roaster. Boxes represent the interquartile range (IQR) between the first and third quartiles, and the line inside represents the median (second quartile). Whiskers denote the lowest and the highest values within 1.56 IQR from the first and third quartiles, respectively. Circles represent outliers beyond the whiskers.

During hot air roasting, the moisture was initially removed from the surface, inducing a water diffusion from the interior to the dried surface. Hot air roasting is a convective heat transfer process, and the temperature rises as a function of heat transfer. As the nut temperature approaches 120 °C, the rate of temperature increase slows and the moisture evaporation accelerates [27]. In the present study, static hot air treatment generally induced a higher detoxification with increasing exposure time. In particular, for Turkish hazelnuts, a higher decontamination of AFB_1_ and AFG_1_ was registered after 40 min of treatment (Figure 1). Italian hazelnuts showed a higher concentration range, but AFB_1_ was always more decontaminated than the other AFs.

With infrared rays, heat is transferred by radiation, and the wavelength determines the temperature. During this type of heating, surface irregularities have a small effect on heating transfer [28]. After infrared rays treatments, residual aflatoxins were lower than 5% and 15%, respectively, on Italian and Turkish hazelnuts. Turkish hazelnuts showed a high decontamination of AFG_1_ for both exposure times and roasting methods, with the lowest value at 40 min treatment. For both methods, a higher reduction of AFB_1_ and AFG_1_ compared to AFB_2_ and AFG_2_ was confirmed. Infrared rays roasting showed better AFs reduction compared to static hot air roasting, due to higher heat transfer efficiency.

### 2.3. Analysis of Perisperm

**Figure 2:**
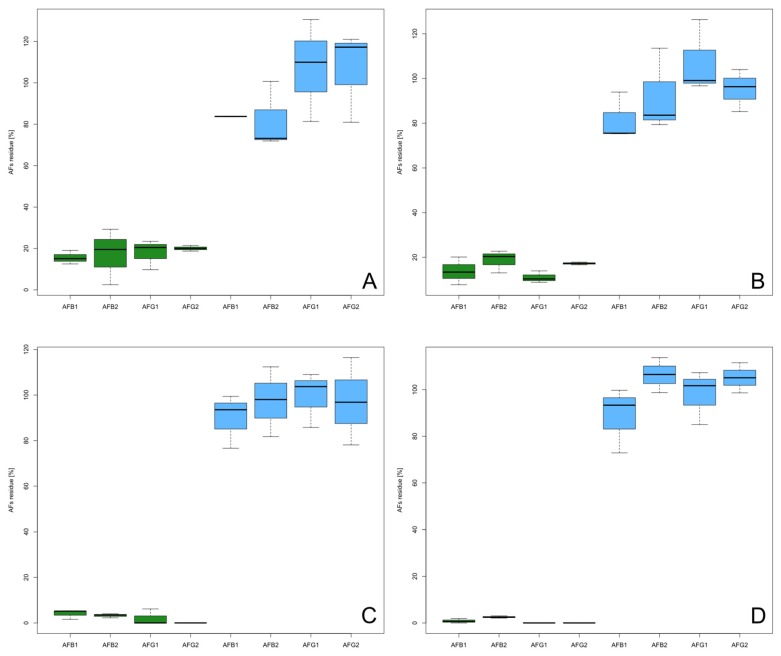
Residual AFB_1_, AFB_2_, AFG_1_, and AFG_2_ on hazelnuts (green) and perisperm (blue). (**A**) 120 °C for 20 min with static hot air roaster; (**B**) 120 °C for 40 min with static hot air roaster; (**C**) 140 °C for 20 min with infrared rays roaster; (**D**) 140 °C for 40 min with infrared rays roaster. All treatments were applied on TGT hazelnuts. Boxes and whiskers meaning is described in Figure 1.

A third set of experiments was realized to understand if the AFs were removed with the perisperm or if they were degraded after exposure to high temperature (Figure 2). The two roasting methods were used only on Italian hazelnuts, and two temperatures (120 °C and 140 °C) were used for 20 and 40 min. After treatments, perisperm was detached from hazelnut and collected separately.

Infrared rays roasting confirmed a lower level for the four AFs (<10%) similar at 20 and 40 min of exposure. Static hot air treatment also showed a similar trend at both exposure times, but the final residue was higher. The perisperm analysis revealed a high concentration of aflatoxins: 72%–126% for the static hot air method, and 73%–114% for infrared rays roasting.

## 3. Conclusions

Both treatments were useful for the detoxification of AFs in hazelnuts without affecting the nutritional properties. After roasting, minor changes occurred in FAs, but the FAs profile was preserved, and unsaturated fatty acids were maintained at high levels. Hot static air roasting induced a detoxification in both types of hazelnuts, with some differences due to the temperature. AFB_1_ and AFG_1_ were always lower than AFB_2_ and AFG_2_ after roasting. When both heating methods were tested under the same conditions, infrared rays roasting induced a higher decontamination. Both thermic treatments were unable to degrade AFs which were accumulated in the perisperm. In conclusion, infrared rays roasting could be considered a promising method for aflatoxin detoxification during hazelnut processing.

## 4. Materials and Methods

### 4.1. Chemicals

LC-MS grade methanol, acetonitrile, and acetic acid used as mobile phases and as extraction solvents were purchased from Sigma-Aldrich (St. Louis, MO, USA). NaCl, KCl, Na_2_HPO_4_, and KH_2_PO_4_ used to prepare phosphate-buffered saline (PBS) solution were purchased from Merck (Darmstadt, Germany) and dissolved in ultrapure water (Maina, Turin, Italy). Pure standards of AFB_1_ (purity ≥ 98%), AFB_2_ (purity ≥ 98%), AFG_1_ (purity ≥ 98%), and AFG_2_ (purity ≥ 98%) were purchased from Sigma-Aldrich. AflaClean select immunoaffinity columns (IAC) were obtained from LCTech (Dorfen, Germany). A working solution of AFs at the concentration of 1 mg/mL was prepared in methanol and diluted in order to contaminate hazelnuts and to prepare calibration curves.

### 4.2. Samples and Contamination Procedure

Hazelnuts used in this work came from Italy and Turkey. The Italian cultivar, “Tonga Gentile Trilobata” (TGT) was harvested in Cortemilia, northern Italy, and provided by La Gentile s.r.l. Turkish hazelnuts were a blend of three major cultivars—“Tombul”, “Palaz”, and “Kalinkara”—from the Ordu region, northern Turkey. All the hazelnuts used were free from aflatoxins. All samples were divided in two parts—one for detoxification experiments and the other for quality analyses; all the hazelnuts were stored in plastic bags, in the dark, at low relative humidity and a temperature of 4 °C before the experiments.

Dehulled raw hazelnuts were artificially contaminated by spraying at the final concentration of 100 ng/g for each of the four aflatoxins in the first set of experiments. In the second set of experiments, the nuts were contaminated with 8 ng/g of AFB_1_ and 2 ng/g of the other three AFs (14 ng/g for total aflatoxins).

### 4.3. Roasters and Other Equipment

Hazelnuts were roasted at the Brovind–GBV s.r.l. company (Cortemilia, CN, Italy) using different equipment. Known quantities of contaminated hazelnuts were roasted in two ovens at specific temperatures and for selected times.

Hot air static roaster model SD-80. The SD-80 static hot air roaster is a laboratory batch roaster, which does not include product mixing and stirring, and hazelnuts are placed on a perforated grill. Roasting is performed by forced circulation of air heated through electric resistance. Working parameters are set up from the master control panel.

Infrared rays roaster model RI/800. The RI/800 roasting oven is a discontinuous laboratory batch machine equipped with an infrared rays heating system. Through a conical hopper, the raw material is introduced into the roasting chamber. Hazelnuts are heated through a battery of IR lamps, and gentle stirring and mixing is supplied by a vibrating tray, which guarantees the homogeneity of the process. After roasting, the product is expelled through a gate into the cooling unit. Working parameters (temperature, time, and vibration) are set from the master control panel.

Cooler/peeler model PR/1. The PR/1 cooler/peeler is made of a circular tank. Hazelnuts are gently stirred by mixing blades, which guarantee a uniform cooling, and nuts are peeled by friction. Cooling is guaranteed by an aspirating collector and an electro-fan with flux regulator.

Pellicle extractor model CSACK/1. The CSACK/1 pellicle aspirator is used to extract powders and fragments of perisperm with a centrifugal electro-fan and store them in a bag.

### 4.4. Treatments

In accordance with the commercial hazelnut treatments, in the first experimental set, two temperatures were chosen for the experiments with the static hot air roaster (120 °C and 170 °C), and two exposure times (20 and 40 min) were applied for each temperature.

In the second experimental set, a new roasting technology—infrared rays roasting—was compared to static hot air roasting. One temperature (140 °C) and two exposure times (20 min and 40 min) were used for both methods. First and second experiments were performed on Italian and Turkish hazelnuts.

A third trial was developed with both roasting methods only on Italian hazelnuts, at the same temperatures (120 °C and 140 °C) and exposure times (20 and 40 min) already tested. After treatments, the hazelnut perisperm was collected separately for the analysis of aflatoxin contents.

### 4.5. Fatty Acid Composition

Fatty acid methyl esters (FAMEs) were determined by gas chromatography according to the method described by Ficarra et al. [29] with slight modification.

Briefly, 50 mg of oil was mixed thoroughly with 1 mL of hexane and 300 μL of 2 M KOH in methanol (*w*/*v*) in a dark tube. The extract was then transferred into a dark glass vial and immediately analyzed by GC. Profiling of the FAMEs was determined using a GC-2010 Shimadzu gas chromatograph (Shimadzu, Kyoto, Japan) equipped with a flame ionization detector, split-splitless injector, an AOC-20i autosampler, and a SP-2560 capillary column (100 m × 0.25 mm id × 0.20 μm, Supelco, Milan, Italy). The following temperature program was used: the initial oven temperature was 165 °C, increasing to 200 °C at 3 °C/min, and then the temperature was held at 200 °C for 45 min. The injector temperature and the detector were 250 °C. Each fatty acid methyl ester was identified and quantified by comparing retention times with Supelco 37 components FAME mix 10 mg/mL. The fatty acid concentration was expressed in relative percentage of each fatty acid, calculated by internal normalization of the chromatographic peak areas. The obtained fatty acid composition was used to calculate the sum of the saturated (∑ SFA), monounsaturated (∑ MUFA), and polyunsaturated (∑ PUFA) fatty acids, as well as the ratio (∑ MUFA + ∑ PUFA)/(∑ SFA). Moreover, to evaluate the oxidative stability, the ratio of oleic to linoleic (O/L) and the iodine value (IV) were determined. The IV was determined according to the percentages of fatty acids using the following formula: (palmitoleic acid × 1.901) + (oleic acid × 0.899) + (linoleic acid × 1.814) + (linolenic acid × 2.737). Significant differences (*p* < 0.05) among the means were determined using a one-way analysis of variance (ANOVA) with Duncan’s test at a fixed level of a = 0.05. All calculations were performed with the STATISTICA software for Windows (Release 7.0; StatSoft Inc., Tulsa, OK, USA).

### 4.6. Extraction, Clean-Up, and LC-MS/MS Conditions

The AFs were extracted and analyzed using the validated method described by Prelle et al. [30]. The analyses were performed on twenty-five grams of ground hazelnuts. For perisperm analysis, 2 g hazelnut skins, obtained after roasting, were extracted for 2 h on a shaker at 165 rpm with 10 mL of mixture solution (methanol:water; 80:20). Samples were centrifuged at 4000 rpm and subsequently filtered by using a Whatman CA 0.45 µm syringe filter (Maldstone, UK). Filtrate was diluted 1:4 in phosphate buffer solution (PBS) for clean-up using IAC. All samples were analyzed in triplicate.

Analyses were performed using a Varian Model 212-LC micro pumps (Palo Alto, CA, USA) with a Varian autosampler Model 410 Prostar coupled with a Varian 310-MS triple quadrupole mass spectrometer with an electrospray ion source operating in positive ionization mode. Chromatographic separation was performed with a Zorbax Eclipse Plus C18 (100 mm × 4.6 mm, 3.8 µm particle size, Agilent, Santa Clara, CA, USA) column using H_2_O and CH_3_OH as eluents, both acidified with 0.1% CH_3_COOH at the flow rate of 0.2 mL/min. Monitoring reaction mode (MRM) transitions used for quantification were: 313 > 285 (CE 14 V) for AFB_1_, 315 > 287 (CE 18 V) for AFB_2_, 329 > 243 (CE 18 V) for AFG_1_, 331 > 245 (CE 24 V) for AFG_2_. AFs quantification was performed using external calibration based on serial dilution of a multi-analyte stock solution.

## Figures and Tables

**Table 1:** Residual (%) aflatoxin B_1_ (AFB_1_), aflatoxin B_2_ (AFB_2_), aflatoxin G_1_ (AFG_1_), and aflatoxin G_2_ (AFG_2_) after treatment with static hot air in the first set of experiments.

Hazelnut	*T* (°C)	*t* (min)	AFB_1_	AFB_2_	AFG_1_	AFG_2_
Italian	120	20	17.5 ± 4.3	17.5 ± 3.8	5.62 ± 2.9	9.46 ± 3.8
Turkish			47.2 ± 2.4	81.7 ± 5.1	77.8 ± 10	69.9 ± 6.0
Italian	120	40	11.7 ± 1.4	13.9 ± 2.5	17.8 ± 5.4	22.5 ± 6.0
Turkish			12.9 ± 8.0	53.1 ± 4.9	12.0 ± 5.2	13.9 ± 5.5
Italian	170	20	38.7 ± 7.7	62.9 ± 5.8	24.7 ± 5.4	68.9 ± 14
Turkish			7.39 ± 1.8	42.3 ± 8.4	10.4 ± 1.9	13.6 ± 5.3
Italian	170	40	17.9 ± 7.3	43.6 ± 10	8.21 ± 1.1	34.5 ± 8.3
Turkish			4.09 ± 4.2	31.3 ± 6.6	6.04 ± 8.5	5.97 ± 8.4

Mean of three replicates ± standard deviation.

**Table 2:** Fatty acid composition (%), of Tonga Gentile Trilobata (TGT) hazelnuts raw and roasted with static hot air and infrared rays.

Fatty Acid	Raw	140 °C 20 min	140 °C 40 min	Significance
**Static Hot Air Treatment**				
Myristic (C14:0)	0.02 ± 0.00	0.02 ± 0.00	0.02 ± 0.00	ns
Palmitic (C16:0)	5.79 ± 0.00 ^b^	5.92 ± 0.01 ^a^	5.74 ± 0.01 ^a^	***
Palmitoleic (C16:1)	0.24 ± 0.00 ^b^	0.24 ± 0.00 ^c^	0.24 ± 0.00 ^a^	***
Margaric (C17:0)	0.04 ± 0.00	0.04 ± 0.00	0.04 ± 0.00	ns
Heptadecenoic (C17:1)	0.07 ± 0.00 ^a^	0.07 ± 0.00 ^b^	0.07 ± 0.00 ^b^	***
Stearic (C18:0)	2.32 ± 0.00 ^c^	2.36 ± 0.00 ^a^	2.27 ± 0.00 ^b^	***
Elaidic (C18:1 ω9t)	0.02 ± 0.00	0.02 ± 0.00	0.02 ± 0.00	ns
Oleic (C18:1 ω9c)	84.86 ± 0.00 ^c^	84.44 ± 0.02 ^b^	84.35 ± 0.03 ^a^	***
Linoleic (C18:2 ω6c)	6.27 ± 0.00 ^a^	6.53 ± 0.01 ^b^	6.88 ± 0.02 ^c^	***
Arachidic (C20:0)	0.11 ± 0.00	0.11 ± 0.00	0.11 ± 0.00	ns
Eicosenoic (C20:1)	0.12 ± 0.00 ^a^	0.12 ± 0.00 ^b^	0.12 ± 0.00 ^c^	***
α-Linolenic (C18:3 ω3)	0.08 ± 0.00 ^b^	0.08 ± 0.00 ^a^	0.08 ± 0.00 ^a^	*
Docosanoic (C22:0)	0.02 ± 0.00 ^ab^	0.02 ± 0.00 ^b^	0.02 ± 0.00 ^a^	*
Arachidonic (C20:4 ω6)	0.03 ± 0.00 ^a^	0.03 ± 0.00 ^a^	0.03 ± 0.00 ^b^	*
∑ SFA	8.31 ± 0.00 ^c^	8.48 ± 0.01 ^a^	8.20 ± 0.01 ^b^	***
∑ MUFA	85.31 ± 0.00 ^c^	84.89 ± 0.02 ^b^	84.80 ± 0.03 ^a^	***
∑ PUFA	6.38 ± 0.00 ^a^	6.64 ± 0.01 ^b^	6.99 ± 0.02 ^c^	***
UFA/SFA	11.03 ± 0.00 ^a^	10.80 ± 0.01 ^c^	11.19 ± 0.02 ^b^	***
Oleic/Linoleic (O/L)	13.54 ± 0.01 ^c^	12.93 ± 0.02 ^b^	12.26 ± 0.03 ^a^	***
Iodine value (IV)	88.34 ± 0.00 ^a^	88.43 ± 0.01 ^b^	88.99 ± 0.01 ^c^	***
**Infrared Rays Treatment**				
Myristic (C14:0)	0.02 ± 0.00	0.02 ± 0.00	0.02 ± 0.00	ns
Palmitic (C16:0)	5.79 ± 0.00 ^b^	6.16 ± 0.02 ^b^	5.94 ± 0.01 ^a^	*
Palmitoleic (C16:1)	0.24 ± 0.00 ^b^	0.27 ± 0.00 ^a^	0.25 ± 0.00 ^b^	***
Margaric (C17:0)	0.04 ± 0.00	0.04 ± 0.00	0.04 ± 0.00	ns
Heptadecenoic (C17:1)	0.07 ± 0.00	0.07 ± 0.00	0.07 ± 0.00	ns
Stearic (C18:0)	2.32 ± 0.00 ^b^	2.44 ± 0.00 ^a^	2.34 ± 0.00 ^b^	*
Elaidic (C18:1 ω9t)	0.02 ± 0.00	0.02 ± 0.00	0.02 ± 0.00	ns
Oleic (C18:1 ω9c)	84.86 ± 0.00 ^a^	84.28 ± 0.03 ^b^	84.44 ± 0.02 ^a^	***
Linoleic (C18:2 ω6c)	6.27 ± 0.00 ^b^	6.32 ± 0.01 ^a^	6.50 ± 0.01 ^b^	***
Arachidic (C20:0)	0.11 ± 0.00	0.12 ± 0.00	0.12 ± 0.00	ns
Eicosenoic (C20:1)	0.12 ± 0.00	0.12 ± 0.00	0.12 ± 0.00	ns
α-Linolenic (C18:3 ω3)	0.08 ± 0.00 ^b^	0.08 ± 0.00 ^a^	0.08 ± 0.00 ^b^	***
Docosanoic (C22:0)	0.02 ± 0.00	0.02 ± 0.00	0.02 ± 0.00	ns
Arachidonic (C20:4 ω6)	0.03 ± 0.00 ^b^	0.03 ± 0.00 ^a^	0.03 ± 0.00 ^b^	***
∑ SFA	8.31 ± 0.00 ^b^	8.81 ± 0.02 ^a^	8.48 ± 0.01 ^b^	*
∑ MUFA	85.31 ± 0.00 ^a^	84.76 ± 0.03 ^b^	84.91 ± 0.01 ^a^	***
∑ PUFA	6.38 ± 0.00 ^b^	6.44 ± 0.01 ^a^	6.62 ± 0.01 ^b^	***
UFA/SFA	11.03 ± 0.00 ^a^	10.36 ± 0.02 ^b^	10.80 ± 0.01 ^a^	*
Oleic/Linoleic (O/L)	13.54 ± 0.01 ^a^	13.34 ± 0.03 ^b^	12.99 ± 0.02 ^a^	***
Iodine value (IV)	88.34 ± 0.00	87.97 ± 0.00 ^a^	88.40 ± 0.00 ^b^	*

Values are expressed as mean ± standard deviation (*n* = 3). Means followed by different letters were significantly different at *p* < 0.05. Where letters in columns were not reported, no statistical differences were observed. Significance: * *p* < 0.05; *** *p* < 0.001; ns = not significant. SFA: saturated fatty acid; MUFA: monounsaturated fatty acid; PUFA: polyunsaturated fatty acid.

**Table 3:** Fatty acid composition (%) of Turkish hazelnut raw and roasted with static hot air and infrared rays.

Fatty Acid	Raw	140 °C 20 min	140 °C 40 min	Significance
**Static** **Hot Air Treatment**				
Myristic (C14:0)	0.03 ± 0.00 ^b^	0.03 ± 0.00 ^a^	0.03 ± 0.00 ^b^	***
Palmitic (C16:0)	5.57 ± 0.00 ^a^	5.58 ± 0.00 ^a^	5.76 ± 0.01 ^b^	***
Palmitoleic (C16:1)	0.17 ± 0.00 ^a^	0.17 ± 0.00 ^b^	0.18 ± 0.00 ^c^	***
Margaric (C17:0)	0.04 ± 0.00 ^a^	0.04 ± 0.00 ^a^	0.04 ± 0.00 ^b^	***
Heptadecenoic (C17:1)	0.07 ± 0.00 ^a^	0.07 ± 0.00 ^b^	0.07 ± 0.00 ^a^	***
Stearic (C18:0)	2.21 ± 0.00 ^c^	2.04 ± 0.00 ^a^	2.17 ± 0.00 ^b^	***
Elaidic (C18:1 ω9t)	0.02 ± 0.00	0.02 ± 0.00	0.02 ± 0.00	ns
Oleic (C18:1 ω9c)	84.87 ± 0.01 ^b^	85.44 ± 0.01 ^c^	84.55 ± 0.01 ^a^	***
Linoleic (C18:2 ω6c)	6.64 ± 0.01 ^b^	6.25 ± 0.00 ^a^	6.81 ± 0.01 ^c^	***
Arachidic (C20:0)	0.12 ± 0.00 ^b^	0.11 ± 0.00 ^a^	0.12 ± 0.00 ^b^	***
Eicosenoic (C20:1)	0.15 ± 0.01	0.14 ± 0.00	0.13 ± 0.00	ns
α-Linolenic (C18:3 ω3)	0.06 ± 0.00 ^a^	0.06 ± 0.00 ^b^	0.07 ± 0.00 ^c^	***
Docosanoic (C22:0)	0.02 ± 0.00	0.02 ± 0.00	0.02 ± 0.00	ns
Arachidonic (C20:4 ω6)	0.03 ± 0.00	0.03 ± 0.00	0.03 ± 0.00	ns
∑ SFA	8.00 ± 0.00 ^b^	7.83 ± 0.01 ^a^	8.15 ± 0.00 ^c^	***
∑ MUFA	85.28 ± 0.01 ^b^	85.83 ± 0.01 ^c^	84.95 ± 0.01 ^a^	***
∑ PUFA	6.73 ± 0.01 ^b^	6.34 ± 0.00 ^a^	6.91 ± 0.01 ^c^	***
UFA/SFA	11.51 ± 0.00 ^b^	11.78 ± 0.01 ^c^	11.27 ± 0.00 ^a^	***
Oleic/Linoleic (O/L)	12.78 ± 0.02 ^b^	13.68 ± 0.01 ^c^	12.42 ± 0.01 ^a^	***
Iodine value (IV)	88.83 ± 0.03 ^a^	88.64 ± 0.00 ^c^	88.87 ± 0.00 ^b^	***
**Infrared** **Rays Treatment**				
Myristic (C14:0)	0.03 ± 0.00 ^b^	0.03 ± 0.00 ^a^	0.03 ± 0.00 ^b^	***
Palmitic (C16:0)	5.57 ± 0.00 ^c^	5.63 ± 0.01 ^b^	5.36 ± 0.04 ^a^	***
Palmitoleic (C16:1)	0.17 ± 0.00 ^b^	0.17 ± 0.00 ^b^	0.16 ± 0.00 ^a^	***
Margaric (C17:0)	0.04 ± 0.00 ^b^	0.04 ± 0.00 ^a^	0.04 ± 0.00 ^a^	***
Heptadecenoic (C17:1)	0.07 ± 0.00 ^a^	0.07 ± 0.00 ^b^	0.07 ± 0.00 ^a^	**
Stearic (C18:0)	2.21 ± 0.00 ^c^	2.08 ± 0.00 ^a^	2.19 ± 0.02 ^b^	***
Elaidic (C18:1 ω9t)	0.02 ± 0.00 ^a^	0.02 ± 0.00 ^ab^	0.03 ± 0.00 ^b^	**
Oleic (C18:1 ω9c)	84.87 ± 0.01	85.08 ± 0.02	84.96 ± 0.58	ns
Linoleic (C18:2 ω6c)	6.64 ± 0.01 ^b^	6.52 ± 0.00 ^c^	6.10 ± 0.04 ^a^	***
Arachidic (C20:0)	0.12 ± 0.00 ^b^	0.11 ± 0.00 ^a^	0.12 ± 0.00 ^c^	***
Eicosenoic (C20:1)	0.15 ± 0.01	0.14 ± 0.00	0.14 ± 0.00	ns
α-Linolenic (C18:3 ω3)	0.06 ± 0.00 ^a^	0.06 ± 0.00 ^c^	0.06 ± 0.00 ^b^	***
Docosanoic (C22:0)	0.02 ± 0.00 ^b^	0.02 ± 0.00 ^a^	0.02 ± 0.00 ^b^	***
Arachidonic (C20:4 ω6)	0.03 ± 0.00	0.03 ± 0.00	0.03 ± 0.00	ns
∑ SFA	8.00 ± 0.00 ^b^	7.90 ± 0.01 ^a^	7.76 ± 0.06 ^a^	***
∑ MUFA	85.28 ± 0.01	85.48 ± 0.02	85.35 ± 0.59	ns
∑ PUFA	6.73 ± 0.01 ^b^	6.61 ± 0.00 ^c^	6.19 ± 0.05 ^a^	***
UFA/SFA	11.51 ± 0.00 ^a^	11.65 ± 0.02 ^b^	11.79 ± 0.01 ^a^	***
Oleic/Linoleic (O/L)	12.78 ± 0.02 ^b^	13.05 ± 0.01 ^a^	13.92 ± 0.01 ^c^	***
Iodine value (IV)	88.83 ± 0.03 ^b^	88.81 ± 0.01 ^c^	87.91 ± 0.61 ^a^	***

Values are expressed as mean ± standard deviation (*n* = 3). Means followed by different letters were significantly different at *p* < 0.05. Where letters in columns were not reported, no statistical differences were observed. Significance: ** *p* < 0.01; *** *p* < 0.001; ns = not significant.

## Supplementary Materials

The following are available online at www.mdpi.com/2072-6651/9/2/72/s1, Table S1: Fatty acid distribution (%) of TGT hazelnuts before and after treatment with static hot air at two temperatures (120 °C and 170 °C) at the two different exposure times (20 and 40 min), Table S2: Fatty acid distribution (%) of Turkish hazelnuts before and after treatment with static hot air at two temperatures (120 °C and 170 °C) at the two different exposure times (20 and 40 min).
