# Supplementary material for: Static Hot Air and Infrared Rays Roasting are Efficient Methods for Aflatoxin Decontamination on Hazelnuts
Source: Toxins (Basel). 2017 Feb 21;9(2):72. doi: 10.3390/toxins9020072 (PMC5331451; doi:10.3390/toxins9020072)
Supplement: Supplementary file 1 [file toxins-09-00072-s001.pdf]

# Supplementary Materials:Static Hot Air and Infrared Rays Roasting are Efficient Methods for Aflatoxin Decontamination on Hazelnuts

**Ilenia Siciliano, Barbara Dal Bello, Giuseppe Zeppa, Davide Spadaro and Maria Lodovica Gullino**

**Table S1.** Fatty acid composition (%) of Tonga Gentile Trilobata (TGT) hazelnut raw and roasted with static hot air at two temperatures (120 °C and 170 °C) at the two different exposure times (20 and 40 min).

| Fatty acid | Raw | 120°C/20 min | 120°C/40 min | 170°C/20 min | 170°C/40 min | Significance |
|---|---|---|---|---|---|---|
| Myristic (C14:0) | 0.03 ± 0.00 [c] | 0.03 ± 0.00 [ab] | 0.03 ± 0.00 [c] | 0.03 ± 0.00 [ab] | 0.03 ± 0.00 [b] | *** |
| Palmitic (C16:0) | 7.04 ± 0.00 [c] | 6.20 ± 0.01 [a] | 6.33 ± 0.01 [c] | 6.51 ± 0.26 [ab] | 6.67 ± 0.29 [b] | *** |
| Palmitoleic (C16:1) | 0.34 ± 0.00 [c] | 0.26 ± 0.00 [a] | 0.26 ± 0.00 [c] | 0.29 ± 0.03 [ab] | 0.31 ± 0.03 [bc] | *** |
| Margaric (C17:0) | 0.05 ± 0.00 | 0.05 ± 0.00 | 0.04 ± 0.00 | 0.05 ± 0.00 | 0.04 ± 0.00 | ns |
| Heptadecenoic (C17:1) | 0.07 ± 0.00 [b] | 0.07 ± 0.00 [b] | 0.06 ± 0.00 [b] | 0.07 ± 0.00 [c] | 0.07 ± 0.00 [bc] | *** |
| Stearic (C18:0) | 2.48 ± 0.00 | 2.51 ± 0.01 | 2.63 ± 0.00 | 2.49 ± 0.13 | 2.44 ± 0.13 | ns |
| Elaidic (C18:1 ω9t) | 0.02 ± 0.00 | 0.02 ± 0.00 | 0.02 ± 0.00 | 0.02 ± 0.00 | 0.02 ± 0.00 | ns |
| Oleic (C18:1 ω9c) | 83.16 ± 0.00 [a] | 84.38 ± 0.01 [b] | 83.74 ± 0.00 [a] | 83.72 ± 0.04 [ab] | 83.71 ± 0.02 [a] | *** |
| Linoleic (C18:2 ω6c) | 6.44 ± 0.00 [b] | 6.08 ± 0.01 [a] | 6.50 ± 0.00 [b] | 6.43 ± 0.16 [b] | 6.32 ± 0.17 [b] | *** |
| Arachidic (C20:0) | 0.12 ± 0.00 [a] | 0.14 ± 0.00 [c] | 0.13 ± 0.00 [a] | 0.13 ± 0.00 [bc] | 0.12 ± 0.01 [bc] | *** |
| Eicosenoic (C20:1) | 0.12 ± 0.00 [a] | 0.14 ± 0.00 [d] | 0.13 ± 0.00 [a] | 0.13 ± 0.00 [c] | 0.13 ± 0.00 [bc] | *** |
| α-Linolenic (C18:3 ω3) | 0.06 ± 0.00 [a] | 0.07 ± 0.00 [c] | 0.07 ± 0.00 [a] | 0.07 ± 0.00 [b] | 0.06 ± 0.00 [b] | *** |
| Docosanoic (C22:0) | 0.02 ± 0.00 [a] | 0.02 ± 0.00 [c] | 0.02 ± 0.00 [a] | 0.02 ± 0.00 [c] | 0.02 ± 0.00 [bc] | *** |
| Arachidonic (C20:4 ω6) | 0.03 ± 0.00 [a] | 0.04 ± 0.00 [bc] | 0.04 ± 0.00 [a] | 0.04 ± 0.00 [cd] | 0.04 ± 0.00 [d] | *** |
| ∑ SFA | 9.75 ± 0.00 [c] | 8.95 ± 0.01 [a] | 9.18 ± 0.00 [c] | 9.23 ± 0.14 [b] | 9.33 ± 0.15 [b] | *** |
| ∑ MUFA | 83.70 ± 0.00 [a] | 84.87 ± 0.01 [c] | 84.21 ± 0.00 [a] | 84.24 ± 0.05 [b] | 84.24 ± 0.02 [b] | *** |
| ∑ PUFA | 6.53 ± 0.00 [b] | 6.19 ± 0.02 [b] | 6.61 ± 0.00 [b] | 6.53 ± 0.16 [b] | 6.43 ± 0.17 [b] | *** |
| UFA/SFA | 9.26 ± 0.00 [a] | 10.18 ± 0.01 [c] | 9.89 ± 0.00 [a] | 9.84 ± 0.16 [b] | 9.72 ± 0.17 [b] | *** |
| Oleic/Linoleic (O/L) | 12.91 ± 0.00 [a] | 13.87 ± 0.03 [b] | 12.88 ± 0.01 [a] | 13.03 ± 0.34 [ab] | 13.24 ± 0.34 [a] | *** |
| Iodine value (I/V) | 87.24 ± 0.00 [a] | 87.57 ± 0.01 [b] | 87.76 ± 0.01 [a] | 87.66 ± 0.25 [b] | 87.49 ± 0.26 [ab] | * |

Values are expressed as mean ± standard deviation ($n$=3). Means followed by different letters were significantly different at $p < 0.05$. Where letters in columns were not reported, no statistical differences were observed. Significance: * $p < 0.05$; *** $p < 0.001$; ns = not significant. SFA: saturated fatty acid; MUFA: monounsaturated fatty acid; PUFA: polyunsaturated fatty acid.

**Table S2.** Fatty acid composition (%) of Turkish hazelnut raw and roasted with static hot air at two temperatures (120 °C and 170 °C) at the two different exposure times (20 and 40 min).

| Fatty acid | Raw | 120°C/20 min | 120°C/40 min | 170°C/20 min | 170°C/40 min | Significance |
|---|---|---|---|---|---|---|
| Myristic (C14:0) | 0.02 ± 0.00 [a] | 0.03 ± 0.00 [b] | 0.03 ± 0.00 [b] | 0.03 ± 0.00 [c] | 0.03 ± 0.00 [b] | *** |
| Palmitic (C16:0) | 4.62 ± 0.00 | 5.01 ± 0.38 | 5.00 ± 0.38 | 5.21 ± 0.00 | 5.22 ± 0.02 | ns |
| Palmitoleic (C16:1) | 0.13 ± 0.00 | 0.14 ± 0.02 | 0.14 ± 0.02 | 0.14 ± 0.00 | 0.14 ± 0.00 | ns |
| Margaric (C17:0) | 0.05 ± 0.00 [a] | 0.05 ± 0.00 [a] | 0.05 ± 0.00 [a] | 0.05 ± 0.00 [b] | 0.05 ± 0.00 [b] | ** |
| Heptadecenoic (C17:1) | 0.07 ± 0.00 | 0.07 ± 0.00 | 0.07 ± 0.00 | 0.07 ± 0.00 | 0.07 ± 0.00 | ns |
| Stearic (C18:0) | 2.34 ± 0.02 [a] | 2.35 ± 0.04 [a] | 2.35 ± 0.04 [a] | 2.39 ± 0.02 [a] | 2.50 ± 0.02 [b] | *** |
| Elaidic (C18:1 ω9t) | 0.02 ± 0.00 [a] | 0.02 ± 0.00 [a] | 0.02 ± 0.00 [a] | 0.02 ± 0.00 [a] | 0.03 ± 0.00 [b] | *** |
| Oleic (C18:1 ω9c) | 85.35 ± 0.02 [b] | 83.96 ± 0.88 [a] | 83.97 ± 0.88 [a] | 84.31 ± 0.02 [a] | 83.88 ± 0.03 [a] | * |
| Linoleic (C18:2 ω6c) | 7.03 ± 0.01 | 8.01 ± 1.23 | 8.01 ± 1.24 | 7.39 ± 0.01 | 7.72 ± 0.02 | ns |
| Arachidic (C20:0) | 0.11 ± 0.00 [a] | 0.11 ± 0.00 [bc] | 0.11 ± 0.00 [de] | 0.12 ± 0.00 [e] | 0.11 ± 0.00 [cd] | *** |
| Eicosenoic (C20:1) | 0.14 ± 0.00 | 0.14 ± 0.01 | 0.14 ± 0.01 | 0.14 ± 0.00 | 0.13 ± 0.00 | ns |
| $\alpha$-Linolenic (C18:3 ω3) | 0.08 ± 0.00 [a] | 0.08 ± 0.00 [a] | 0.08 ± 0.00 [a] | 0.09 ± 0.00 [c] | 0.09 ± 0.00 [b] | *** |
| Docosanoic (C22:0) | 0.02 ± 0.00 [a] | 0.02 ± 0.00 [a] | 0.02 ± 0.00 [ab] | 0.02 ± 0.00 [b] | 0.02 ± 0.00 [a] | * |
| Arachidonic (C20:4 ω6) | 0.02 ± 0.00 [b] | 0.02 ± 0.00 [ab] | 0.01 ± 0.00 [a] | 0.02 ± 0.00 [b] | 0.02 ± 0.00 [b] | *** |
| ∑ SFA | 7.16 ± 0.03 [a] | 7.56 ± 0.34 [ab] | 7.56 ± 0.34 [ab] | 7.82 ± 0.02 [b] | 7.93 ± 0.02 [b] | ** |
| ∑ MUFA | 85.71 ± 0.02 [b] | 84.32 ± 0.89 [a] | 84.33 ± 0.89 [a] | 84.68 ± 0.02 [a] | 84.25 ± 0.03 [a] | * |
| ∑ PUFA | 7.13 ± 0.01 | 8.11 ± 1.23 | 8.11 ± 1.23 | 7.50 ± 0.01 | 7.82 ± 0.02 | ns |
| UFA/SFA | 12.97 ± 0.05 [b] | 12.24 ± 0.58 [a] | 12.24 ± 0.58 [a] | 11.79 ± 0.03 [a] | 11.61 ± 0.04 [a] | *** |
| Oleic/Linoleic (O/L) | 12.13 ± 0.01 | 10.68 ± 1.93 | 10.68 ± 1.93 | 11.40 ± 0.01 | 10.87 ± 0.03 | ns |
| Iodine value (IV) | 89.96 ± 0.03 [a] | 90.51 ± 1.41 [b] | 90.51 ± 1.42 [b] | 89.73 ± 0.01 [c] | 89.90 ± 0.03 [b] | ns |

Values are expressed as mean ± standard deviation ($n$=3). Means followed by different letters were significantly different at $p < 0.05$. Where letters in columns were not reported, no statistical differences were observed. Significance: * $p < 0.05$; ** $p < 0.01$; *** $p < 0.001$; ns = not significant.
